# A powerful score-based test statistic for detecting gene-gene co-association

**DOI:** 10.1186/s12863-016-0331-3

**Published:** 2016-01-29

**Authors:** Jing Xu, Zhongshang Yuan, Jiadong Ji, Xiaoshuai Zhang, Hongkai Li, Xuesen Wu, Fuzhong Xue, Yanxun Liu

**Affiliations:** Department of Biostatistics, School of Public Health, Shandong University, 44 Wen Hua Xi Road, PO Box 100, Jinan, 250012 China; Department of Epidemiology and Statistics, Bengbu Medical College at Bengbu, Anhui, 233030 China

**Keywords:** Gene-gene co-association, Score-based, Gene-based

## Abstract

**Background:**

The genetic variants identified by Genome-wide association study (GWAS) can only account for a small proportion of the total heritability for complex disease. The existence of gene-gene joint effects which contains the main effects and their co-association is one of the possible explanations for the “missing heritability” problems. Gene-gene co-association refers to the extent to which the joint effects of two genes differ from the main effects, not only due to the traditional interaction under nearly independent condition but the correlation between genes. Generally, genes tend to work collaboratively within specific pathway or network contributing to the disease and the specific disease-associated locus will often be highly correlated (e.g. single nucleotide polymorphisms (SNPs) in linkage disequilibrium). Therefore, we proposed a novel score-based statistic (SBS) as a gene-based method for detecting gene-gene co-association.

**Results:**

Various simulations illustrate that, under different sample sizes, marginal effects of causal SNPs and co-association levels, the proposed SBS has the better performance than other existed methods including single SNP-based and principle component analysis (PCA)-based logistic regression model, the statistics based on canonical correlations (CCU), kernel canonical correlation analysis (KCCU), partial least squares path modeling (PLSPM) and delta-square (*δ*^2^) statistic. The real data analysis of rheumatoid arthritis (RA) further confirmed its advantages in practice.

**Conclusions:**

SBS is a powerful and efficient gene-based method for detecting gene-gene co-association.

**Electronic supplementary material:**

The online version of this article (doi:10.1186/s12863-016-0331-3) contains supplementary material, which is available to authorized users.

## Background

Genome-wide association study (GWAS) has successfully identified numerous loci associated with complex disease or traits [1–3]. Despite high expectations, one common sense is that the genetic variants identified by GWAS can only account for a small proportion of the total heritability for complex disease, referring to “missing heritability” problem [4–6]. Possible explanations for this problem include the existence of gene-gene joint effects, the contribution of rare variation, underestimation of the effects of alleles identified, the possibility that inherited epigenetic factors lead to resemblance between relatives and possible overestimation of heritability of the interested complex disease or traits [4–7]. It is highly desirable to further develop more efficient statistical strategies to extract more information from the high-throughput data. Among these, one key but inadequately addressed issue is the joint effects of two genes, which contains the main effects and their co-association.

**Fig. 1:**
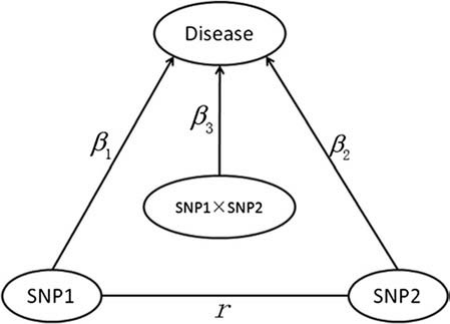
A causal graph for two SNPs affecting the disease

Our group has proposed the concept of gene-gene co-association which refers to the extent to which the joint effects of two genes differs from the main effects of each gene in previous studies [8–11]. The distinction between gene-gene co-association and interaction has been theoretically clarified from the causal diagram perspective [9], and various simulations have also been conducted to confirm its reasonability, especially for two highly correlated genes. Specifically, taking 2 SNPs as an example (Fig. 1), the main effects of SNP1 and SNP2 are supposed to be *β*_1_ and *β*_2_ respectively and the correlation coefficient between them is *r*. The total effects of SNP1 and SNP2 are denoted as *β*_1_ + *β*_2_ + *β*_3_ + *r*(*β*_1_ + *β*_2_) and the term *β*_3_ + *r*(*β*_1_ + *β*_2_) represents the co-association where the traditional interaction *β*_3_is only one part of co-association [9]. Actually, gene-gene co-association is essentially used to capture the joint effects attributed to the correlation *r*(*β*_1_ + *β*_2_), which has usually been neglected in traditional regression model. Generally, genes tend to work collaboratively within specific pathway or network that is associated with certain disease [12–15] and the disease-associated interacting locus will often be highly correlated (single nucleotide polymorphisms (SNPs) in linkage disequilibrium (LD)) [16]. In this context, gene-gene co-association should be more appropriate to cope with the missing heritability problem. On the other hand, testing the co-association of two genes can, to some extent, guide us to learn and construct genetic network structures. It is of great significance to develop methods for detecting gene-gene co-association.

Recently, several methods have been proposed to test gene-gene co-association, such as the statistics based on SNP-level Fisher r-to-z transformation [9], canonical correlation analysis (CCU) [8], kernel canonical correlation analysis (KCCU) [11] and partial least squares path modeling (PLSPM) [10]. SNP-level Fisher r-to-z transformation-based statistics, though having acceptable false positive rates and computation burden, fail to fully utilize the LD information between markers and true causal SNPs in one gene or region, leading to lower statistical power. Furthermore, single SNP can hardly represent the total effect of the whole gene on a disease. It is appealing to construct gene or region-based statistics to detect gene-gene co-association, such as the latter three statistics including CCU, KCCU and PLSPM-based statistics. However, CCU statistic [8] merely captures linear correlation which may be inappropriate for genomic data containing nonlinear structure, and it only utilizes the first canonical correlation coefficient, which may underestimate the gene-gene co-association. Although KCCU statistic [11], as the nonlinear version of CCU, can detect the nonlinear information, it still remains the uncertainty to set the kernel function with appropriate parameters for each testing data leading to undesirable performance, as well as the high computational burden due to the use of bootstrap test. Similarly, PLSPM-based statistic [10] can deal with the problems of high multicollinearity between SNPs, but it is also time-consuming resulting from the employment of random permutation test. Therefore, developing powerful and efficient gene-based methods to test gene-gene co-association is highly desirable.

At present study, we aimed to develop a powerful score-based test statistic to identify co-association at gene or region level, which essentially captured the effect of covariance matrix between two genes on disease. Various simulation studies were conducted to assess its type I error rate and power, comparing with the commonly-used single SNP-based logistic regression model (SNP-LRT) [17–19], principle component analysis (PCA)-based logistic regression model (PCA-LRT) [20], the delta-square (*δ*^2^) statistic [16], the CCU statistic [8], the KCCU statistic [11] and the PLSPM-based statistic [10]. Finally, the proposed score-based statistic (SBS) was applied to analyze a rheumatoid arthritis (RA) data from GAW16 Problem 1. Both simulation and real data analysis indicate that the proposed statistic has better performance than other existing methods.

## Methods

### Score-based Statistic

We denote *Y*_*i*_ as observed binary trait outcome of individual *i*(*i* = 1, 2, …, *n*) in the GWAS data set and let the genotype data be (*X*_11_, *X*_12_, …, *X*_1*k*_, …, *X*_1*K*_) for gene A with *K* SNPs and (*X*_21_, *X*_22_, …, *X*_2*j*_, …, *X*_2*J*_) for gene B with *J* SNPs. Particularly, for the *k*^*th*^ loci of gene A and *j*^*th*^ loci of gene B, we can firstly define the variability score for each sample by ${u}_{kji}=\left({X}_{1ki}-{\overline{X}}_{1k}\right)\left({X}_{2ji}-{\overline{X}}_{2j}\right)$, where ${\overline{X}}_{1k}$ and ${\overline{X}}_{2j}$ indicate the mean level of *k*^*th*^ loci of gene A and *j*^*th*^ loci of gene B respectively. Then, the score-based statistic for their co-association effect can be defined as ${u}_{kj}={\displaystyle {\sum}_{i=1}^n\left({Y}_i-\overline{Y}\right)}\left({X}_{1ki}-{\overline{X}}_{1k}\right)\left({X}_{2ji}-{\overline{X}}_{2j}\right)$, where $\overline{Y}$ is the sample mean of disease status. Furthermore, the score vector with the length of K*J can be defined as *U* = (*u*_11_, *u*_12_, …, *u*_1*K*_, *u*_21_, …, *u*_2*K*_, …, *u*_*kj*_, …, *u*_*K*1_, …, *u*_*KJ*_), and covariance matrix for the score vector can be easily obtained as

$$ \varSigma =\left(\begin{array}{l}cov\left({u}_{11},{u}_{11}\right),cov\left({u}_{11},{u}_{12}\right),cov\left({u}_{11},{u}_{13}\right),\dots, cov\left({u}_{11},{u}_{kj}\right),\dots, cov\left({u}_{11},{u}_{KJ}\right)\\ {}cov\left({u}_{12},{u}_{11}\right),cov\left({u}_{12},{u}_{12}\right),cov\left({u}_{12},{u}_{13}\right),\dots, cov\left({u}_{12},{u}_{kj}\right),\dots, cov\left({u}_{12},{u}_{KJ}\right)\\ {}\vdots \\ {}cov\left({u}_{kj},{u}_{11}\right),cov\left({u}_{kj},{u}_{12}\right),cov\left({u}_{kj},{u}_{13}\right),\dots, cov\left({u}_{kj},{u}_{kj}\right),\dots, cov\left({u}_{kj},{u}_{KJ}\right)\\ {}\vdots \\ {}cov\left({u}_{KJ},{u}_{11}\right),cov\left({u}_{KJ},{u}_{12}\right),cov\left({u}_{KJ},{u}_{13}\right),\dots, cov\left({u}_{KJ},{u}_{kj}\right),\dots, cov\left({u}_{KJ},{u}_{KJ}\right)\end{array}\right) $$

Finally, the new score-based statistic for detecting gene-gene co-association can be constructed as *SBS* = *UΣ*^− 1^*U*^*T*^, which follows chi-square distribution with K*J degree freedom (*χ*_*K* ∗ *J*_^2^) under the null hypothesis that there is no co-association between these two genes.

### Data simulation

Simulation studies were conducted to assess the type I error rate and power of the SBS comparing with other methods for testing gene-gene co-association. We simulated three co-association scenarios as follows: Type I co-association (under nearly independent condition between gene A and gene B, i.e. the traditional interaction *β*_3_), Type II co-association (only caused by correlation between gene A and gene B, i.e. *r*(*β*_1_ + *β*_2_)),Type III co-association (caused by both correlation and independent term *A* × *B* between gene A and gene B, i.e. *β*_3_ + *r*(*β*_1_ + *β*_2_)). Specifically, the null hypothesis for all three simulation scenarios can be described as inexistence of co-association between two genes. Reference phased haplotype data was downloaded from the *HapMap* website (http://hapmap.ncbi.nlm.nih.gov/) [21]. Subsequently, a large CEU population of 100,000 individuals was obtained by gs2.0 [22, 23] under the additive genetic model. In all simulations, the causal SNPs were removed to assess the performances of the SBS. For each parameter setting, 1000 simulations were repeated with a significant level of 0.05 and *N* individuals were sampled from the whole 100,000 population randomly.

For scenario 1 (Type I co-association), we chose 7 SNPs at Chr17:1650000215…1650011216 and 7 SNPs at Chr18:1700258917…1700276475. The case-control statuses were generated from a logistic regression model *Logit*(*P*) = *β*_0_ + *β*_1_ × *SNP*_1_ + *β*_2_ × *SNP*_2_ + *β*_3_ × (*SNP*_1_ × *SNP*_2_), where SNP1 and SNP2, correlated with coefficient *r* were causal SNPs, and the 1^*st*^ SNP of gene A and 5^*th*^ SNP of gene B were defined as the causal SNPs. Three different main effects were set to make our simulations more practical, two marginal effects (*β*_1_ = *log*(1.3), *β*_2_ = *log*(1.5)), one marginal effect (*β*_1_ = 0, *β*_2_ = *log*(1.5)) and no marginal effects (*β*_1_ = *β*_2_ = 0). Different *β*_3_ were chosen to evaluate the type I error rate (*r* = 0, *β*_3_ = 0) under various sample sizes *N* (*N*/2 cases and *N*/2 controls, *N* = 400, …, 2000) and power (*β*_3_ was specified from *log*(1.1) to *log*(1.9) stepped by *log*(0.2)) under fixed sample size 1200. In addition, we also fixed the interaction odds ratio and main effects to assess the performance of the SBS under different sample sizes.

For scenario 2 (Type II co-association), we chose 7 SNPs at Chr22:2126161008…2126164539 and 7 SNPs at Chr22:2126166075…2126177318. In this situation, the case-control statuses were generated from the logistic regression model *Logit*(*P*) = *β*_0_ + *β*_1_ × *SNP*_1_ + *β*_2_ × *SNP*_2_. Different *r* were specified to evaluate the type I error rate (*β*_1_ = *β*_2_ = *β*_3_ = 0, *r* = 0.1, 0.2, 0.3, 0.4, 0.5, 0.9) and power under fixed main effects *β*_1_ = 0, *β*_2_ = *log*(1.5) and *β*_1_ = *log*(1.3), *β*_2_ = *log*(1.5) for the two causal SNPs with given sample size 1200. To evaluate the performance under different MAF of causal SNP pairs, different correlation structures between two causal SNPs were chosen from the two regions.

For scenario 3 (Type III co-association), we selected the same gene region as in the scenario 2. The case-control statuses were generated from the model *Logit*(*P*) = *β*_0_ + *β*_1_ × *SNP*_1_ + *β*_2_ × *SNP*_2_ + *β*_3_ × (*SNP*_1_ × *SNP*_2_). Two situations were considered: *β*_3_ was specified from *log*(1.1) to *log*(1.9) stepped by *log*(0.2) under fixed *r*, and *r* was set from 0.1to 0.5 by 0.1under fixed *β*_3_. All the simulations were conducted under sample size 1200 and different main effect patterns (*β*_1_ = *β*_2_ = 0, *β*_1_ = 0, *β*_2_ = *log*(1.5) and *β*_1_ = *log*(1.3), *β*_2_ = *log*(1.5)).

For the single SNP-based logistic regression model,we considered each pair-wise interaction separately, and selected the most significant one (smallest *p*-values). Significane levels were assessed using permutations to adjust the multiple testing [10].

### Applications

The SBS was also applied to a GWAS of North American Rheumatoid Arthritis (RA) Consortium containing 868 RA cases and 1194 controls [24] and all datasets used were publically available [25, 26]. We chose four genes (*VEGFA, PADI4, C5, ITGAV*) to detect gene-gene co-association with RA susceptibility, involving four, six, eight and eight SNPs in each gene respectively. Meanwhile, the other six methods mentioned above were also used to detect co-association contributing to RA and their computation time was also calculated by R 3.1.0 on a desktop computer (Intel Core 2 with 3.00 GHz CPU using 4 GB of RAM).

## Results

### Simulation

Tables 1 and 2 show the type I error rates of the seven methods for different sample sizes in various scenarios (*β*_1_ = 0, *β*_2_ = *log*(1.5) and *β*_1_ = *log*(1.3), *β*_2_ = *log*(1.5)) under *β*_3_ = *r* = 0, while Table 3 shows the type I error rates under *β*_1_ = *β*_2_ = *β*_3_ = 0, *r* ≠ 0 with the sample size of 1200. It indicates that the type I error rates of all methods are within the acceptable range and more close to the given nominal level 0.05 with the larger sample sizes. Similar results can be obtained under the case (*β*_1_ = *β*_2_ = 0) in Additional file 1.

**Table 1:** The type I error rates of the seven methods without correlation and interaction under (*β*_1_ = *log*(1.3), *β*_2_ = *log*(1.5))

Sample size	SBS	CCU	PCA	PLSPM	Logistic	KCCU	*δ* ^2^
400	0.043	0.024	0.054	0.061	0.050	0.045	0.060
800	0.047	0.045	0.057	0.058	0.048	0.048	0.051
1200	0.045	0.070	0.053	0.055	0.045	0.053	0.047
1600	0.048	0.072	0.058	0.054	0.056	0.056	0.062
2000	0.054	0.053	0.056	0.058	0.047	0.051	0.058

**Table 2:** The type I error rates of the seven methods without correlation and interaction under (*β*_1_ = 0, *β*_2_ = *log*(1.5))

Sample size	SBS	CCU	PCA	PLSPM	Logistic	KCCU	*δ* ^2^
400	0.045	0.023	0.058	0.046	0.049	0.048	0.047
800	0.042	0.037	0.045	0.061	0.047	0.047	0.054
1200	0.044	0.051	0.040	0.062	0.053	0.054	0.059
1600	0.052	0.038	0.043	0.062	0.048	0.051	0.043
2000	0.053	0.041	0.045	0.064	0.049	0.061	0.053

**Table 3:** The type I error rates of the seven methods without main effects and interaction (*β*_1_ = 0, *β*_2_ = 0, *β*_3_ = 0)

r	SBS	CCU	PCA	logistic	PLSPM	KCCU	*δ* ^2^
0.1	0.043	0.054	0.046	0.047	0.044	0.032	0.047
0.2	0.045	0.038	0.056	0.048	0.048	0.037	0.053
0.3	0.048	0.040	0.044	0.048	0.032	0.045	0.055
0.4	0.052	0.035	0.040	0.045	0.064	0.056	0.048
0.5	0.047	0.061	0.046	0.047	0.034	0.048	0.049
0.9	0.046	0.058	0.042	0.046	0.054	0.038	0.044

**Fig. 2:**
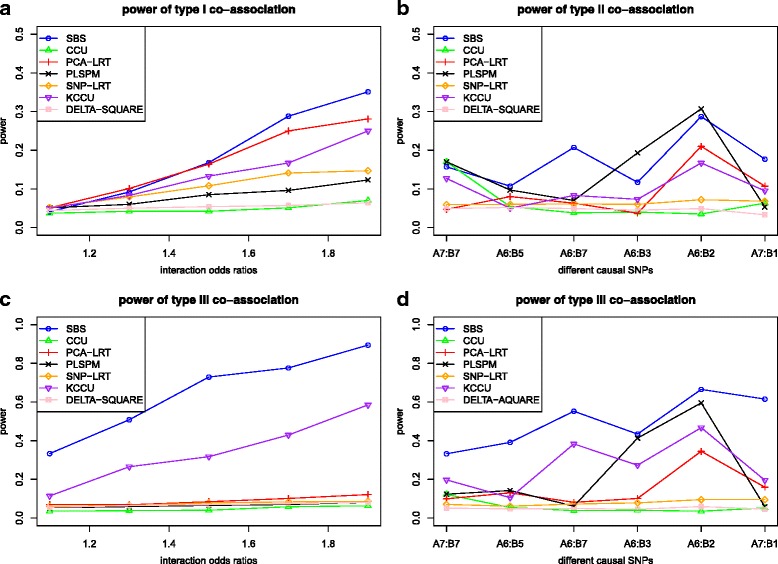
The power of the seven methods under different co-association levels with two main effects (*β*_1_ = *log*(1.3), *β*_2_ = *log*(1.5)). Note: figure (**a**) for Type I co-association with different interaction effects; figure (**b**) for Type II co-association with different causal SNP pairs; figure (**c**) for Type III co-association given fixed correlation 0.3 and different interaction effects; figure (**d**) for Type III co-association given fixed interaction effect *β*_3_ = *log*(1.3) and different causal SNP pairs

The power of the seven methods for type I co-association is shown in Fig. 2a under various interaction effects when *β*_1_ = *log*(1.3), *β*_2_ = *log*(1.5) with sample size 1200. Obviously, the power of most methods increases monotonically as the interaction effects increase, and the SBS shows relatively higher power than the others. Similar power trends as a function of sample sizes also emerged under fixed marginal effects (*β*_1_ = *log*(1.3), *β*_2_ = *log*(1.5)) and interaction effect (*β*_3_ = *log*(1.5)) in Additional file 2.

For type II co-association, the power of the seven methods is shown in Fig. 2b. With the main effects of two genes at 1.3 and 1.5 (*β*_1_ = *log*(1.3), *β*_2_ = *log*(1.5)) and the interaction odds ratio at 1(*β*_3_ = 0), the power of the SBS shows relatively better performance than other methods no matter what the MAF of the two causal SNPs is. Furthermore, under *β*_3_ = 0, Additional file 3: Figure S2 illustrates the power when the summation of the main effects of the two causal SNPs is fixed as *log*(2.8) (see Additional file 3). The proposed SBS shows highest power and all methods show the same trends, indicating that the type II gene-gene co-association can indeed be caused only by correlation, i.e. (*r*(*β*_1_ + *β*_2_)).

Shown in Fig. 2c and d are the results of the power for type III co-association. Figure 2c shows the results under various interaction odds ratios with the correlation coefficient at 0.3 and the sample size 1200. It reveals that the power of the seven methods increase monotonically as the interaction odds ratios increase. Apparently, the SBS outperforms all the other methods. Figure 2d shows the results under various causal SNP pairs with *β*_3_ = *log*(1.3) and the sample size 1200. It indicates that the SBS always keeps the highest power, though the power of all the methods varies heavily under different MAFs. Our proposed SBS is quite suitable for detecting gene-gene co-association under high correlations comparing with other methods.

**Fig. 3:**
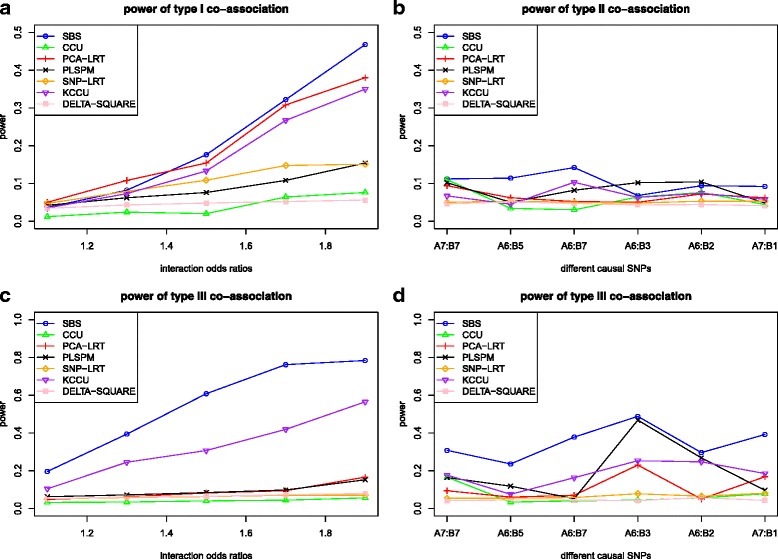
The power of the seven methods under different co-association levels with one main effect (*β*_1_ = 0, *β*_2_ = *log*(1.5)). Note: figure (**a**) for Type I co-association with different interaction effects; figure (**b**) for Type II co-association with different causal SNP pairs; figure (**c**) for Type III co-association given fixed correlation 0.3 and different interaction effects; figure (**d**) for Type III co-association given fixed interaction effect *β*_3_ = *log*(1.3) and different causal SNP pairs

Under the situation with only one main effect (*β*_1_ = 0, *β*_2_ = *log*(1.5)), similar phenomenon also appeared (Fig. 3), except that the power under this situation was a little lower than that under the situation with two main effects. In addition, the results under *β*_1_ = 0, *β*_2_ = 0 further confirmed this in Additional file 4.

### Application

Table 4 shows the results of gene-gene co-association analysis of all seven methods for 868 RA cases and 1194 controls. Our proposed SBS, CCU statistic and KCCU statistic all suggest that co-association of *VEGFA-PADI4* and *C5-PADI4* is significant with RA susceptibility at nominal level 0.05, whereas no significance can be found from the other methods. With regard to the computation time, take the *VEGFA-PADI4* as the example, the computation time for the SBS takes 1.02 s, 3.72 s for CCU, 99.6 s for single SNP-based logistic regression model, 0.6 s for PCA-based logistic regression model, 6.18 s for *δ*^2^ statistic, 26.76 s for PLSPM, while up to 42 h for the KCCU using the same desktop computer (Intel Core 2 with 3.00 GHz CPU using 4 GB of RAM).

**Table 4:** *P*-values of gene-gene co-association among *VEGFA*, *C5*, *PADI4* and *ITGAV*

Co-association	SBS	CCU	PCA	logistic	PLSPM	KCCU	*δ* ^2^
*VEGFA-PADI4*	0.045*	0.046*	0.383	0.448	0.699	<0.001*	0.729
*C5-PADI4*	0.035*	0.047*	0.804	1.000	0.648	<0.001*	0.579
*ITGAV-PADI4*	0.101	0.141	0.805	1.000	0.636	<0.001*	0.186

*significant at level 0.05

## Discussion

The existence of gene-gene joint effects which contain the main effects and their co-association, is one of the possible explanations for the “missing heritability” problems. Gene-gene co-association refers to the extent to which the joint effects of two genes differ from the main effects, not only due to the traditional interaction under nearly independent condition but the correlation between genes. It is often customarily put into the framework of gene-gene interaction, and is identified by adding the product term into the traditional regression method. However, most diseases are caused by multiple genes acting together through pathways or networks where genes (or SNPs) are often correlated rather independence. The implying independence assumption of the regression model is rarely satisfied and the effects attributed to the correlation have usually been neglected. In addition, when constructing a priori topological structure for establishing genetic networks that contribute to diseases of interest, it seems more reasonable to test whether significant relationships between any two nodes in such networks exist or not by detection for gene-gene co-association rather than traditional interaction. Thus, it is crucial to develop powerful methods to detect gene-gene co-association.

In this paper, we have proposed a powerful score-based statistic for testing gene-gene co-association at gene or region level. One appealing property is that it theoretically has rigorous asymptotic distribution under the null hypothesis, which is computationally efficient without using permutation or bootstrap techniques. Actually, our group had developed several methods to detect gene-gene co-association, such as Fisher r-to-z transformation-based statistics, CCU, KCCU and PLSPM-based statistics. One common disadvantage for these methods is the high computation burden. Furthermore, comparing with other existed methods, several simulations had been conducted to confirm the stability and advantage of the proposed score-based statistic under various co-association scenarios. For type I co-association, the power of the proposed score-based statistic was close to PCA-based logistic regression model under smaller interaction odds ratio. While, as the interaction odds ratio increased, the increasing speed of its power was far beyond the other methods. In addition, under type II co-association and type III co-association, some methods (e.g. CCU statistic) did not work at all since they could not capture the correlation information between causal SNPs. In this context, our proposed score-based statistic still outperformed others. Though the proposed score-based statistic performed a little poorer than PLSPM-based statistic under some situations, its power kept higher than PLSPM under more realistic situations when causal SNP pairs were in stronger correlation. For the real data analysis, our proposed score-based statistic can detect the co-association of *VEGFA-PADI4* and *C5-PADI4* which have been reported earlier [8, 11], and its computation time was relative smaller than that of most methods, though a little larger than that of PCA-based logistic regression. This further confirmed its practicability. In addition, we also compared the proposed score-based statistic with the least absolute shrinkage and selection operator (LASSO) as a classical shrinkage-based method [27]. All the simulation results indicated that the proposed score-based statistic had the better performance than LASSO. It is indeed necessary to provide detailed information about the calculation of P-value. The P-value in LASSO is the proportion of the corresponding coefficients of the product terms greater than 0 among all SNP pairs. For instance, suppose there are 7 SNPs in each gene, we first removed one causal SNP pair to deal with the indirect association, then totally 6 × 6 = 36 product terms of SNP pairs were left and put into the LASSO regression model simultaneously. We recorded the corresponding coefficients which were not equal to 0 as m, and m/36 was calculated as the P-value. Finally, the power was calculated by averaging all the *P*-values from 1000 simulations. The R package *lars* has been used for LASSO in the simulations. We have added the corresponding results into the Additional file 1: Table S2, Table S3 and Additional file 5: Figure S4.

Since our proposed method is developed based on the classical score test, it can be easily extended to analyze gene-gene co-association for continuous traits, which we can similarly calculated the score statistics from likelihood function. It is indeed important to guard against possible heterogeneity caused by some other covariates (e.g. age, gender, smoking status). One possible solution for this is Mantel-Haenszel method, which may suffer small sample size problem when the number of covariates is quite large. Another possible way is to calculate the conditional score statistics given the covariates.

One limitation for the proposed score-based statistic is that it considers all possible SNP pairs from the two genes, and it may fail to rigorously follow the chi-square distribution if the number of SNPs is quite large. At present, it is quite difficult to give some recommendations regarding to the appropriate number of SNPs, since the performance of our proposed statistic depends on the sample sizes, the underlying gene structures and the co-association effects. If the number of SNPs is too large, one possible solution is to adopt the non-parametric methods such as permutation test, another is to determine the tag SNPs from each gene first to reduce the number of SNPs and then to apply our proposed statistic to detect gene-gene co-association. Actually, one natural and most commonly used algorithm for tag SNPs selection is based on the principle of the linkage disequilibrium (LD), where tag SNPs can usually be captured based on two-marker (pairwise) or multimarker measures of LD [28]. In practice, all LD and haplotype block analyses can be achieved by Haploview software [29]. Furthermore, there are many other methods have been recently proposed, including the weighted tag-SNP-set analytical method [30], the CLONTagger method [31], the diSNP selection method [32] and the FastTagger method [33]. Meanwhile, it is inevitable to yield very noisy covariance matrices and face multiple testing problems once extending the proposed statistic to a large genome-wide scale, which should be considered in the future.

## Conclusions

The proposed score-based statistic is a powerful and efficient gene-based method for detecting gene-gene co-association compared to CCU, KCCU, PLSPM-based statistics, *δ*^2^statistic, single SNP-based and PCA-based logistic regression test.

### Availability of supporting data

The GWAS data of North American Rheumatoid Arthritis Consortium were downloaded from the Genetic Analysis Workshop (http://www.gaworkshop.org/) with application in advance.

## Additional files

Additional file 1: Table S1. The type I error rates of the seven methods without correlation and interaction under (*β*_1_ = 0, *β*_2_ = 0, *β*_3_ = 0). **Table S2.** The type I error rates of the SBS and LASSO without correlation and interaction under (*β*_1_ = *log*(1.3), *β*_2_ = *log*(1.5)). **Table S3.** The type I error rates of the SBS and LASSO without main effects and interaction (*β*_1_ = 0, *β*_2_ = 0, *β*_3_ = 0). (DOCX 24 kb) Additional file 2: Figure S1. The power of the seven methods under different sample sizes with two main effects and fixed interaction effect (*β*_1_ = *log*(1.3), *β*_2_ = *log*(1.5), *β*_3_ = *log*(1.5)) for type I co-association. (PDF 3 kb) Additional file 3: Figure S2. The power of the seven methods when the summation of the main effects of the two causal SNPs were fixed as *log*(2.8), interaction effect at *β*_3_ = 0 and the correlation at 0.5 for type II co-association. (PDF 3 kb) Additional file 4: Figure S3. The power of the seven methods under different co-association levels with no main effect (*β*_1_ = 0, *β*_2_ = 0). Note: figure a for Type I co-association with different interaction effects; figure b for Type II co-association with different causal SNP pairs; figure c for Type III co-association given fixed correlation 0.3 and different interaction effects; figure d for Type III co-association given fixed interaction effect *β*_3_ = *log*(1.3) and different causal SNP pairs. (PDF 7 kb) Additional file 5: Figure S4. The power of the SBS and LASSO under different co-association levels with two main effect (*β*_1_ = *log*(1.3), *β*_2_ = *log*(1.5)). Note: figure a for Type I co-association with different interaction effects; figure b for Type II co-association with different causal SNP pairs; figure c for Type III co-association given fixed correlation 0.3 and different interaction effects; figure d for Type III co-association given fixed interaction effect *β*_3_ = *log*(1.3) and different causal SNP pairs. (PDF 3 kb)

## Abbreviations

SBS: Score-based statistic
GWAS: Genome-wide association study
SNPs: Single nucleotide polymorphisms
CCU: Statistic based on canonical correlations
KCCU: Statistic based on kernel canonical correlation analysis
PLSPM: Partial least squares path modeling
PCA: Principle component analysis
LASSO: The least absolute shrinkage and selection operator
LD: Linkage disequilibrium
RA: Rheumatoid arthritis
