# Supplementary material for: A powerful score-based test statistic for detecting gene-gene co-association
Source: BMC Genet. 2016 Jan 29;17:31. doi: 10.1186/s12863-016-0331-3 (PMC4731962; doi:10.1186/s12863-016-0331-3)
Supplement: Additional file 1: Table S1. — The type I error rates of the seven methods without correlation and interaction under (β 1 = 0, β 2 = 0, β 3 = 0). Table S2. The type I error rates of the SBS and LASSO without correlation and interaction under (β 1 = log(1.3), β 2 = log(1.5)). Table S3. The type I error rates of the SBS and LASSO without main effects and interaction (β 1 = 0, β 2 = 0, β 3 = 0). (DOCX 24 kb) [file 12863_2016_331_MOESM1_ESM.docx]

| **Table S1.The type I error rates of the seven methods without correlation and interaction under (**$\beta_{1}=0,\beta_{2}=0$**).** | | | | | | | |
| --- | --- | --- | --- | --- | --- | --- | --- |
| Sample size | SBS | CCU | PCA | PLSPM | logistic | KCCU | $\delta^{2}$ |
| 400 | 0.042 | 0.024 | 0.044 | 0.057 | 0.049 | 0.042 | 0.045 |
| 800 | 0.041 | 0.032 | 0.051 | 0.053 | 0.048 | 0.046 | 0.048 |
| 1200 | 0.048 | 0.054 | 0.047 | 0.049 | 0.052 | 0.054 | 0.043 |
| 1600 | 0.052 | 0.069 | 0.056 | 0.056 | 0.046 | 0.057 | 0.052 |
| 2000 | 0.049 | 0.036 | 0.052 | 0.049 | 0.049 | 0.049 | 0.059 |

| **Table S2.The type I error rates of the SBS and LASSO without correlation and interaction under (**$\beta_{1}=log(1.3),\beta_{2}=log(1.5)$**).** | | | |
| --- | --- | --- | --- |
| Sample size | SBS | LASSO |  |
| 400 | 0.043 | 0.034 |  |
| 800 | 0.047 | 0.057 |  |
| 1200 | 0.045 | 0.047 |  |
| 1600 | 0.048 | 0.052 |  |
| 2000 | 0.054 | 0.046 |  |

**Table S3.The type I error rates of the SBS and LASSO without main effects and interaction (**$\beta_{1}=0,\beta_{2}=0,\beta_{3}=0$**).**

| r | SBS | LASSO |
| --- | --- | --- |
| 0.1 | 0.043 | 0.043 |
| 0.2 | 0.045 | 0.041 |
| 0.3 | 0.048 | 0.043 |
| 0.4 | 0.052 | 0.043 |
| 0.5 | 0.047 | 0.044 |
| 0.9 | 0.046 | 0.044 |
