# Supplementary figures and images for: A powerful score-based test statistic for detecting gene-gene co-association
Source: BMC Genet. 2016 Jan 29;17:31. doi: 10.1186/s12863-016-0331-3 (PMC4731962; doi:10.1186/s12863-016-0331-3)

# power under different sample size of type I co-association

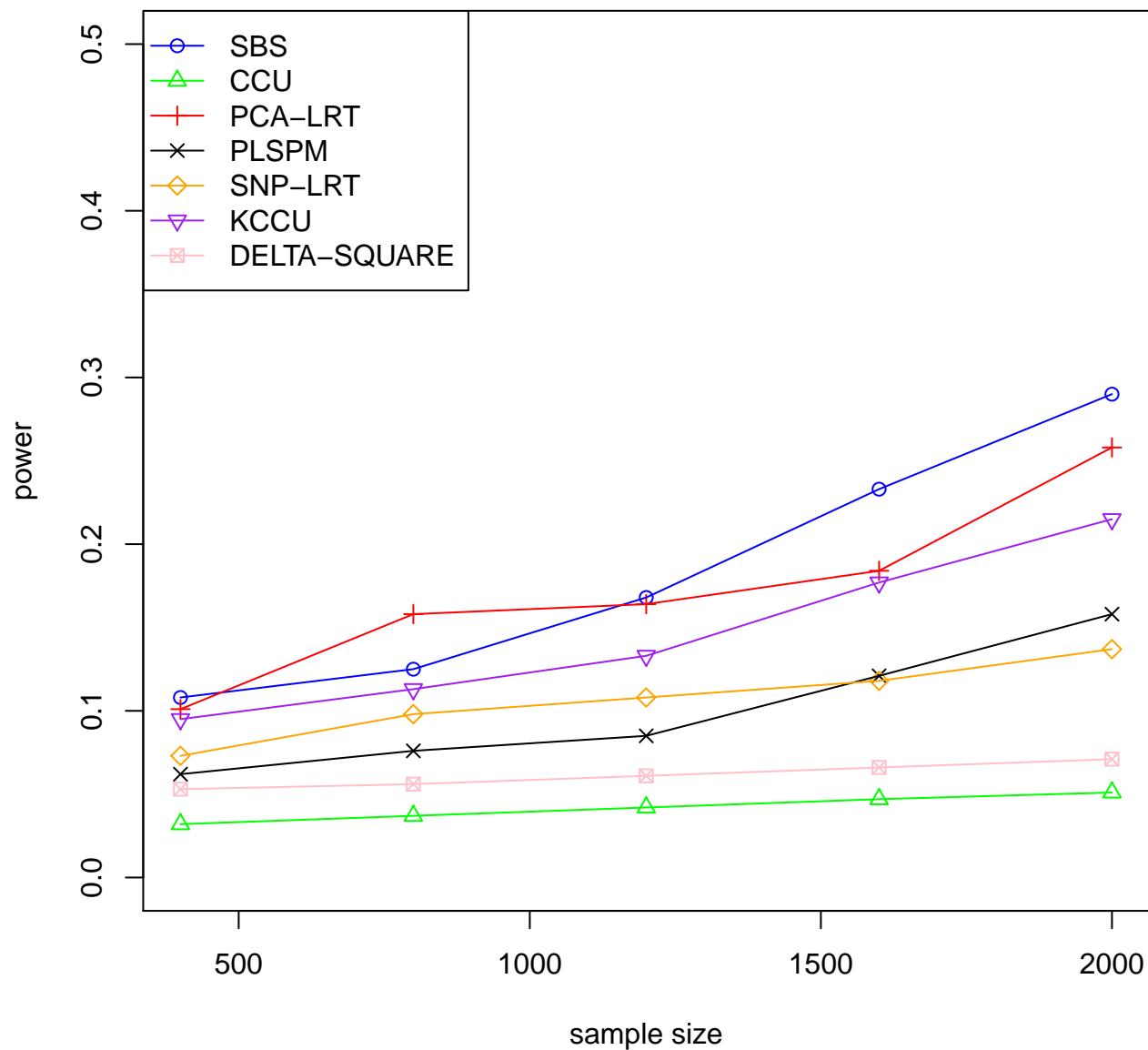

Supplement: Additional file 2: Figure S1. — The power of the seven methods under different sample sizes with two main effects and fixed interaction effect (β 1 = log(1.3), β 2 = log(1.5), β 3 = log(1.5)) for type I co-association. (PDF 3 kb) [file 12863_2016_331_MOESM2_ESM.pdf]

power under different marginal odds ratio pairs of type II co-association

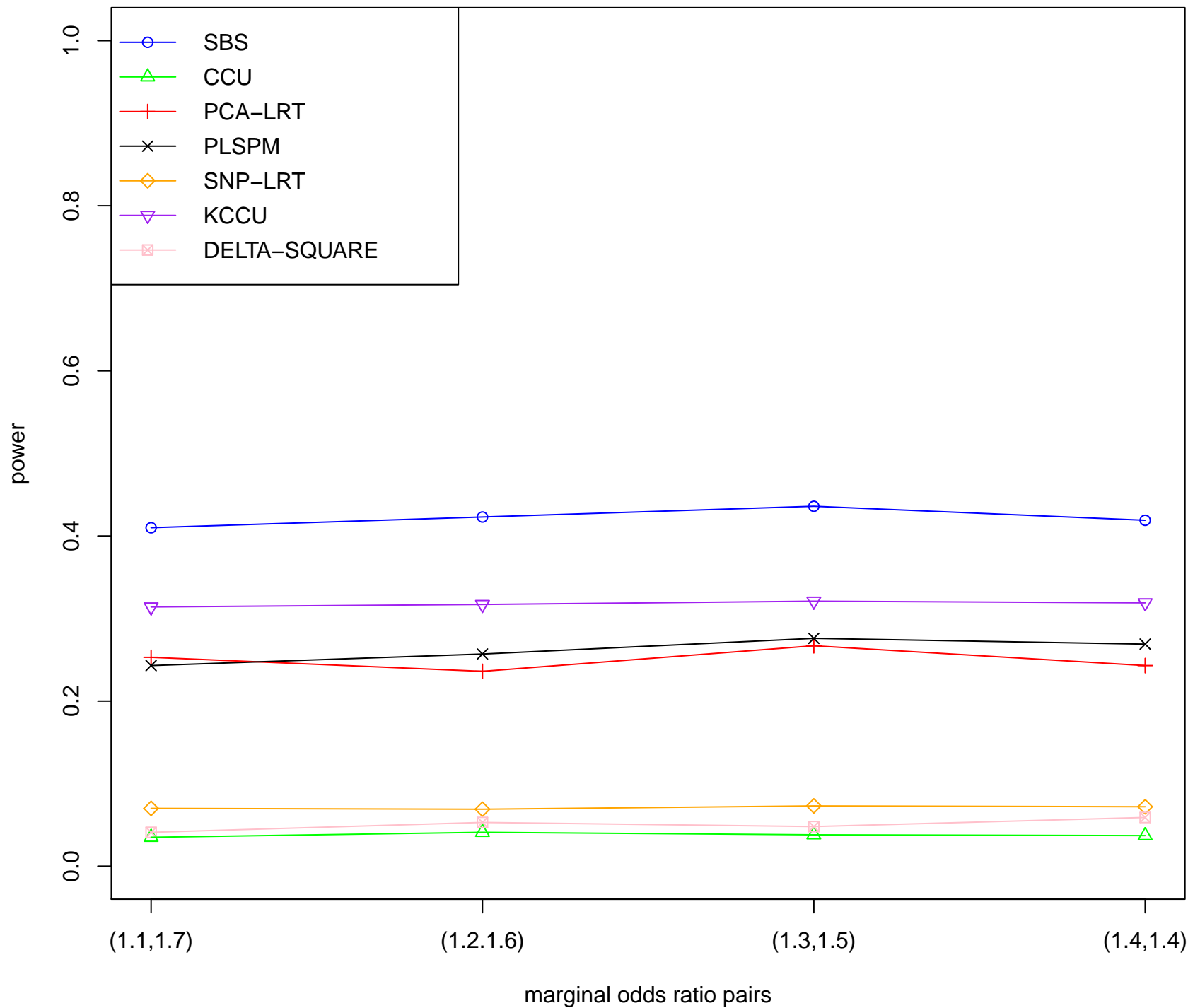

Supplement: Additional file 3: Figure S2. — The power of the seven methods when the summation of the main effects of the two causal SNPs were fixed as log(2.8), interaction effect at β 3 = 0 and the correlation at 0.5 for type II co-association. (PDF 3 kb) [file 12863_2016_331_MOESM3_ESM.pdf]
