# Supplementary material for: A powerful score-based test statistic for detecting gene-gene co-association
Source: BMC Genet. 2016 Jan 29;17:31. doi: 10.1186/s12863-016-0331-3 (PMC4731962; doi:10.1186/s12863-016-0331-3)
Supplement: Additional file 4: Figure S3. — The power of the seven methods under different co-association levels with no main effect (β 1 = 0, β 2 = 0). Note: figure a for Type I co-association with different interaction effects; figure b for Type II co-association with different causal SNP pairs; figure c for Type III co-association given fixed correlation 0.3 and different interaction effects; figure d for Type III co-association given fixed interaction effect β 3 = log(1.3) and different causal SNP pairs. (PDF 7 kb) [file 12863_2016_331_MOESM4_ESM.pdf]

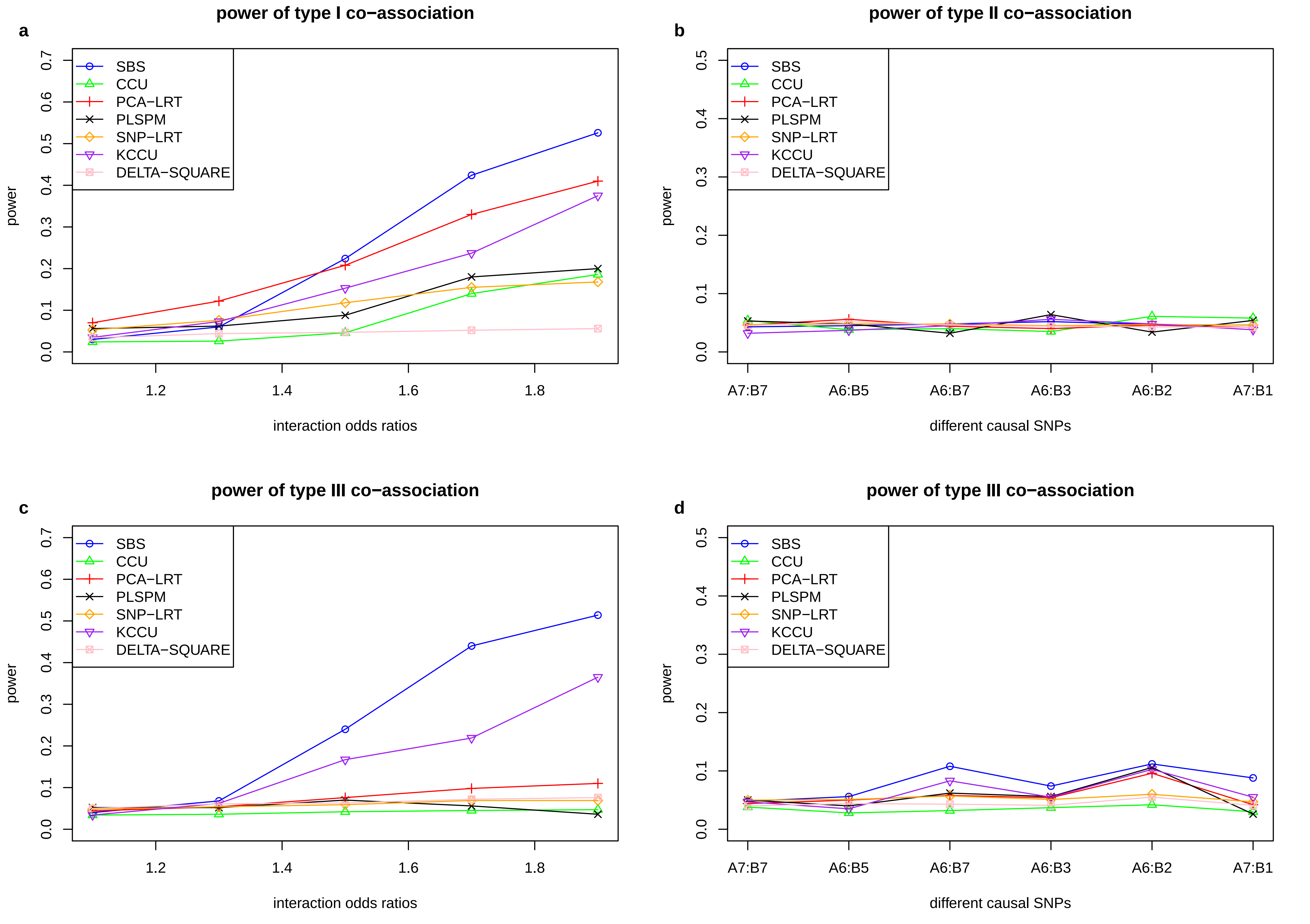

power of type I co−association
a
SBS
CCU
PCA−LRT
PLSPM
SNP−LRT
KCCU
DELTA−SQUARE
power
0.0
0.1
0.2
0.3
0.4
0.5
0.6
0.7
1.2
1.4
1.6
1.8
interaction odds ratios
power of type II co−association
b
SBS
CCU
PCA−LRT
PLSPM
SNP−LRT
KCCU
DELTA−SQUARE
power
0.0
0.1
0.2
0.3
0.4
0.5
A7:B7
A6:B5
A6:B7
A6:B3
A6:B2
A7:B1
different causal SNPs
power of type III co−association
c
SBS
CCU
PCA−LRT
PLSPM
SNP−LRT
KCCU
DELTA−SQUARE
power
0.0
0.1
0.2
0.3
0.4
0.5
0.6
0.7
1.2
1.4
1.6
1.8
interaction odds ratios
power of type III co−association
d
SBS
CCU
PCA−LRT
PLSPM
SNP−LRT
KCCU
DELTA−SQUARE
power
0.0
0.1
0.2
0.3
0.4
0.5
A7:B7
A6:B5
A6:B7
A6:B3
A6:B2
A7:B1
different causal SNPs
